#  Case report: Radiographic complete response of radiation-induced glioblastoma to front-line radiotherapy: A report and molecular characterization of two unique cases

**DOI:** 10.3389/fneur.2023.1099424

**Published:** 2023-03-21

**Authors:** Patrick T. Grogan, Jeffrey J. Helgager, Dustin A. Deming, Steven P. Howard, Robert B. Jenkins, H. Ian Robins

**Affiliations:** ^1^Department of Medicine, Division of Hematology, Medical Oncology, and Palliative Care, University of Wisconsin, Madison, WI, United States; ^2^McArdle Laboratory for Cancer Research, Department of Oncology, University of Wisconsin, Madison, WI, United States; ^3^Carbone Comprehensive Cancer Center, University of Wisconsin, Madison, WI, United States; ^4^Department of Pathology and Laboratory Medicine, University of Wisconsin, Madison, WI, United States; ^5^Department of Human Oncology, University of Wisconsin, Madison, WI, United States; ^6^Department of Laboratory Medicine and Pathology, Mayo Clinic, Rochester, MN, United States; ^7^Department of Neurology, University of Wisconsin, Madison, WI, United States

**Keywords:** radiation-induced glioma (RIG), complete response (CR), radiation, molecular sequencing, chromosomal microarray

## Abstract

Radiation-induced gliomas (RIGs) are an uncommon disease type and a known long-term complication of prior central nervous system radiation exposure, often during childhood. Given the rarity of this malignancy subtype, no clinical trials have explored optimal therapy for these patients, and the literature is primarily limited to reports of patient cases and series. Indeed, the genomic profiles of RIGs have only recently been explored in limited numbers, categorizing these gliomas into a unique subset. Here, we describe two cases of RIG diagnosed as glioblastoma (GB), IDH-wildtype, in adults who had previously received central nervous system radiation for childhood cancers. Both patients demonstrated a surprising complete radiographic response of the postoperative residual disease to front-line therapy, a phenomenon rarely observed in the management of any GB and never previously reported for the radiation-induced subgroup. Both tumors were characterized by next-generation sequencing and chromosomal microarray to identify potential etiologies for this response as well as to further add to the limited literature about the unique molecular profile of RIGs, showing signatures more consistent with diffuse pediatric-type high-grade glioma, *H3*-wildtype, and IDH-wildtype, WHO grade 4. Ultimately, we demonstrate that treatment utilizing a radiation-based regimen for GB in a previously radiated tissue can be highly successful despite historical limitations in the management of this disease.

## Introduction

Radiation remains a staple therapeutic option for many pediatric and adult malignancies. Given the mutagenic nature of this therapy, patients receiving ionizing radiation in the management of their disease early in life are at risk for future treatment-related complications, including secondary malignancies (1, 2). Survivors of childhood cancer, after exposure to radiation and/or chemotherapy, have a 20.5% incidence of secondary malignancies 30 years after the primary diagnosis (3). Specifically, pediatric patients originally treated with radiation, regardless of chemotherapy or initial disease type, demonstrated a significantly increased risk of developing secondary central nervous system (CNS) neoplasms, including gliomas and meningiomas at median latency periods of 9- and 17-year post-radiation, respectively (4).

Radiation-induced gliomas (RIGs) have long been anecdotally thought to act more aggressively than other *de novo* gliomas. Indeed, survival data for this disease—which effectively consist of compilations of case reports and retrospective data—have been correspondingly poor with median overall survival (mOS) times of 17 and 9 months for radiation-induced histologic grade 3 and 4 gliomas, respectively (5). Despite prior tissue exposure to radiation and concern for long-term radiation recall pathology, reirradiation has been safely utilized in these patients. In contrast to chemotherapy and surgery, only radiation has been associated with prolonged survival. Both 1- and 2-year overall survival were comparable between this RIG cohort (58.9, 20.5%) and the radiation alone arm in the original Stupp temozolomide (TMZ) trial (50.6, 10.4%), despite the inclusion of grade 3 disease with GB for the RIG group; histologic grade 3 astrocytoma survival was far inferior to what has been seen in the CATNON trial overall cohort (6, 7). For patients with RIG not managed with radiation, 1-year survival has been reported to be less than 20% (5).

In addition, favorable prognostic markers such as isocitrate dehydrogenase (*IDH*) mutation and methylguanine-O6-methyltransferase (*MGMT*) promotor methylation are less likely to be found in RIG, particularly the former, supporting unfavorable outcomes for radiation-induced GB (8, 9). More recent reports on high-grade RIGs similarly support these abysmal outcomes with mOS ranging from 6.0 to 8.5 months depending on the original pediatric diagnosis (8).

Despite the disappointing survival outcomes in RIG, reirradiation is a meaningful treatment option for some patients based on retrospective and extrapolated data. It remains unclear which patients are more likely to benefit from this therapy. In this regard, while molecular testing, including genomic sequencing, has emerged in neuro-oncology as necessary testing to establish diagnosis, prognosis, and guide therapeutic options, there remains a paucity of this data for RIGs due to its low incidence albeit with emerging evidence suggestive of a “unifying molecular signature” for this disease (8, 10, 11). Herein, we report two cases of aggressively behaving RIGs, diagnosed as GB, IDH-wildtype, based on histologic and molecular criteria using the revised fourth edition of the World Health Organization (WHO) Classifications of CNS Tumors, with exceptional radiographic responses to radiation therapy. We also provide a detailed description of the histologic examination and molecular evaluation of these tissues to contribute to the limited data reported in the literature and touch upon diagnostic considerations for these neoplasms with the new pathologic classification criteria for gliomas in the WHO Classification of CNS Tumors, fifth edition.

## Case presentations

### Case 1

A 49-year-old man with a history of pediatric acute lymphoid leukemia at the age of 4 years, treated with chemoradiation including cranial vault radiation with a midplane dose of 24.12 Gy (spinal radiation status unknown), initially presented to an outside facility with a 3-week history of intractable headaches, relieved by conservative measures, and visual changes described as “seeing fireworks” when his eyes were closed. This improved, but 2 months later, the patient presented to an emergency department for left-sided weakness, fatigue, dizziness, and recurrent headaches with visual changes. An MRI of the head was ultimately completed showing a 5.5 × 5.2 × 4.7 cm posterior parasagittal extra-axial mass with a significant local mass effect and invasion of the superior sagittal sinus and overlying calvarium consistent with a meningioma, presumably radiation-induced. It is noted that there was a 9 × 8 × 8 mm enhancing focus in the left posterior parietal lobe/angular gyrus of unclear etiology but with glioma of consideration. The patient underwent a craniotomy for the resection of the meningioma along with reported embolization at the outside facility; pathology was reported as a grade 2 meningioma.

**Figure 1:**
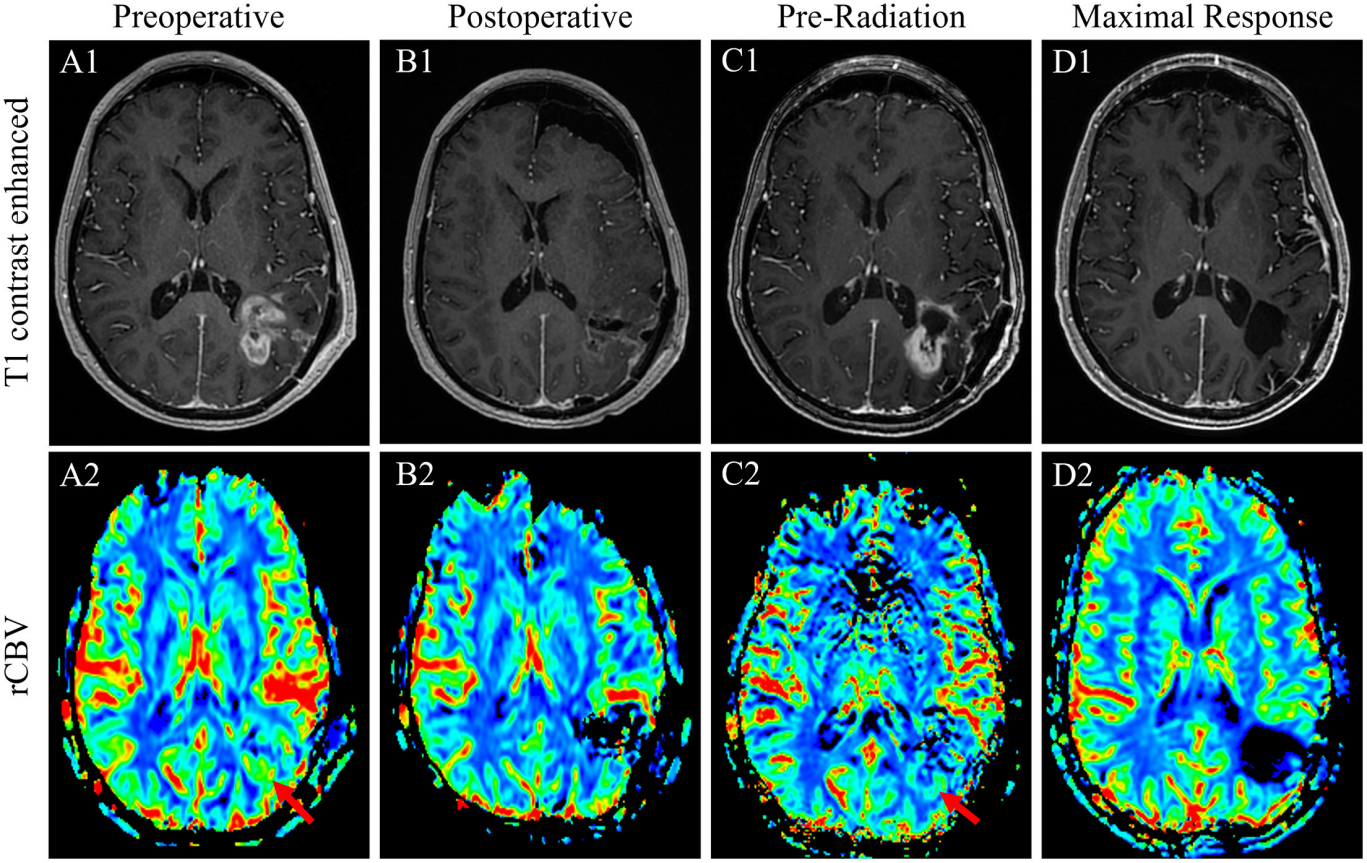
T1-weighted contrasted sequences (1) and relative cerebral blood volume MRI sequences of case 1 patient's GB at the time of initial regrowth and presentation to our facility before the re-resection of the *de novo* disease **(A)**; immediately postoperative result after a near-gross total re-resection **(B)**; 1 week prior to the initiation of chemoradiation with substantial interval disease regrowth appreciated **(C)**; and the time of maximal response with complete disease remission of 6 weeks after the conclusion of radiation therapy and after one cycle of adjuvant TMZ **(D)**.

**Figure 2:**
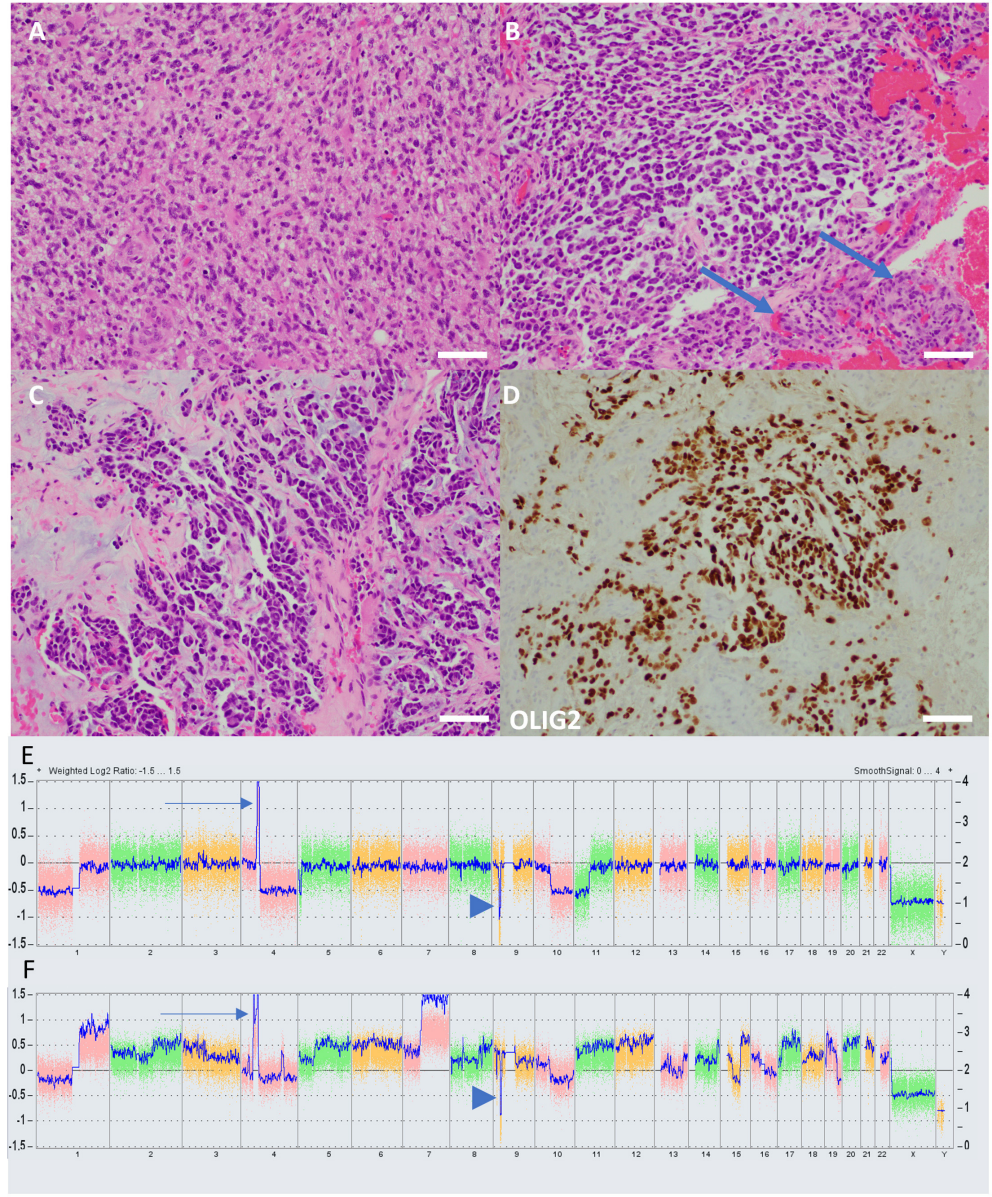
Histology and molecular characteristics of cases. **(A)** The H&E-stained section from case 1 demonstrates histologic features of a typical high-grade diffuse glial neoplasm, including frequent mitoses and anaplasia. Vascular proliferation and necrosis, grading features diagnostic of GB, were present but not shown. **(B)** In contrast, an H&E-stained section from case 2 demonstrates a tumor not immediately reminiscent of glioma, with a prominent myxoid background and cells which take on an epithelioid appearance. Florid vascular proliferation is present (arrows). **(C)** In case 2, the tumor has almost glandular architecture, which is suggestive of metastatic carcinoma. **(D)** An immunostain for OLIG-2, a marker of glial lineage, nonetheless confirms that the tumor in case 2 is, indeed, glioma. The pathologist diagnosing case 2, given the uncharacteristic appearance of the tumor, felt it necessary to run several confirmatory immunohistochemical markers, including cytokeratins, to rule out entities such as metastatic carcinoma and melanoma. Such prominent myxoid changes have been reported previously in radiation-induced gliomas with molecular characteristics of diffuse pediatric high-grade glioma, H3-wildtype, and IDH-wildtype (scale bars = 100 μM). Chromosomal microarray graphical plots of case 1 **(E)** and case 2 **(F)** demonstrating characteristic *PDGRA* amplification (arrow) and *CDKN2A* deep deletion (arrowhead). Case 2 has a considerably more complex karyotype with multiple gains, losses, and amplifications.

Subsequent imaging and management were delayed for unclear reasons, but an MRI of the head was repeated 3 months postoperatively showing a new 3.1 × 3.3 × 2.6 cm heterogeneously enhancing necrotic mass in the left parietal lobe concerning the prior lesion site, concerning a GB. A craniotomy was performed for disease resection but with a postoperative MRI head showing residual tumor for a subtotal resection. Pathology was consistent with glioblastoma, IDH-wildtype, and methylated *MGMT* promotor. The patient sought an opinion at our institution 3 weeks later. An MRI at that time showed substantial enhancing disease regrowth adjacent to the resection cavity, supported by elevated cerebral blood volume and diffusion restriction (Figures 1A1, A2). Given this regrowth, the patient underwent a re-resection 1 week later with a questionable concern for residual disease in the medial resection cavity (Figures 1B1, B2); pathology confirmed the radiographic findings of regrowth and a diagnosis of GB (Figure 2A). Targeted genomic sequencing with StrataNGS revealed *CDKN2A* deep deletion, *KIT* amplification, and *PDGFRA* amplification along with variants of unknown significance in *ALK, BRCA1, JAK1, PDGFRA*, and *XPC*. Chromosomal microarray on the original resection tissue supported the StrataNGS results and demonstrated loss of chromosome (Chr) regions in 1p, 4q, 5p, 9p, 10q, and 11p (Figure 2E).

Unfortunately, an MRI done 3 weeks later again demonstrated substantial tumor regrowth around the resection cavity (Figures 1C1, C2). Given the inability to safely complete a gross total resection and the implications of early regrowth from yet another craniotomy, the decision was made to move forward with chemoradiation. The patient was treated with a modified Stupp regimen with 6 weeks of concurrent TMZ and radiation (60 Gy in 30 fractions) followed by six adjuvant cycles of TMZ. Concurrent TMZ was reduced to 50 mg/m^2^ given data supporting the efficacy of this approach secondary to concerns about inducing radiation necrosis from reirradiation and marrow reserve questions if prior spinal radiation had, indeed, been given (12). An MRI of the head was obtained 6 weeks after the completion of radiation and remarkably demonstrated a complete resolution of the extensive enhancement, associated perfusion, and restricted diffusion previously at the periphery of the left parietal resection cavity (Figures 1D1, D2). The patient went on to complete standard-dose adjuvant TMZ without significant side effects, and an MRI at this time demonstrated the development of subependymal enhancement along the posterior lateral body of the left lateral ventricle without perfusion or restricted diffusion consistent with radiation necrosis. The patient is currently over 24 months from the completion of radiation without evidence of clear disease recurrence.

### Case 2

**Figure 3:**
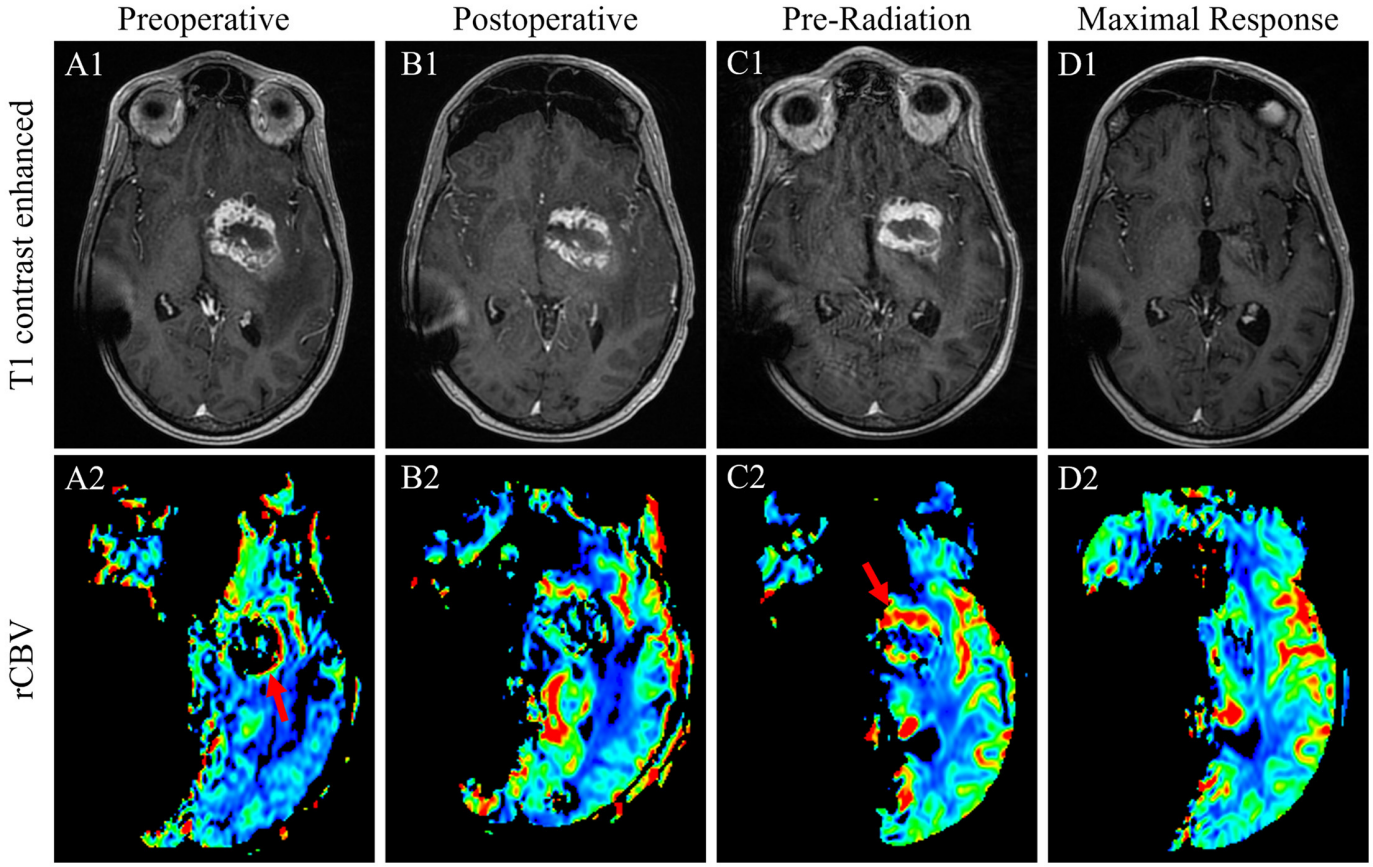
T1-weighted contrasted sequences (1) and relative cerebral blood volume MRI sequences of case 2 patient's GB at the time of initial diagnosis **(A)**; immediate postoperative results following a subtotal resection **(B)**; 1-month postoperative results prior to the initiation of radiation with evidence of substantial persistent disease with the evidence of interval progression **(C)**; and the time of maximal response in the brain with complete disease remission 7 months after the conclusion of radiation therapy **(D)**.

The patient was a 41-year-old man with a history of a non-biopsied pediatric brain tumor thought to most likely be an intracranial germ cell tumor diagnosed at age 13 and treated with craniospinal radiation, complicated by panhypopituitarism and hydrocephalous with a need for a ventriculoperitoneal shunt; specific details of the delivered radiotherapy were unknown. He presented to an outside facility with several days of mild confusion, right-facial droop, right-hand weakness, balance issues, and apparent expressive aphasia (later clarified as anomic aphasia and verbal apraxia) in the setting of traveling to run a marathon. An MRI of the head was completed and demonstrated a 4.5 × 3.4 × 2.9 cm heterogeneously enhancing hemorrhagic mass centered in the left basal ganglia and extending into the left mesial temporal region with significant surrounding T2/FLAIR hyperintense signal, concerning a GB (Figures 3A1, A2). The patient was discharged to our facility where he underwent a craniotomy with subtotal resection 3 days after the initial MRI (Figures 3B1, B2). Pathology was consistent with glioblastoma, IDH-wildtype, and unmethylated *MGMT* promotor (Figures 2B–D). Targeted genomic sequencing with StrataNGS revealed only a *CDKN2A* deep deletion. Chromosomal microarray supported the StrataNGS, identified low-level amplification of *PDGFRA*, and demonstrated a tetraploid pathology with additional site-specific copy number variations (Figure 2F).

One month postoperatively, a pre-therapy MRI of the head revealed increasingly thickened T1 contrast enhancement that was more conspicuous and with new extension posterior to the primary lesion, all demonstrating increased perfusion consistent with postoperative disease growth (Figures 3C1, C2). An MRI of the total spine to rule out drop metastases was unremarkable. Given an ECOG performance status of 4 and complete right hemiplegia postoperatively as well as the lack of *MGMT* promotor methylation, the patient was deemed a poor candidate for standard concurrent TMZ with radiation and underwent hypofractionated intensity-modulated radiotherapy to 40.05 Gy in 15 fractions. Unexpectedly, an MRI of the head 8 weeks after the completion of radiation revealed a marked decrease in residual enhancing tumor and T2/FLAIR signal. The T1 contrast-enhanced tumor further decreased 3 months later and was completely resolved 7 months after the completion of radiation (Figures 3D1, D2). The patient maintained functionality during this time and regained minimal right-sided motor function with intensive therapy.

**Figure 4:**
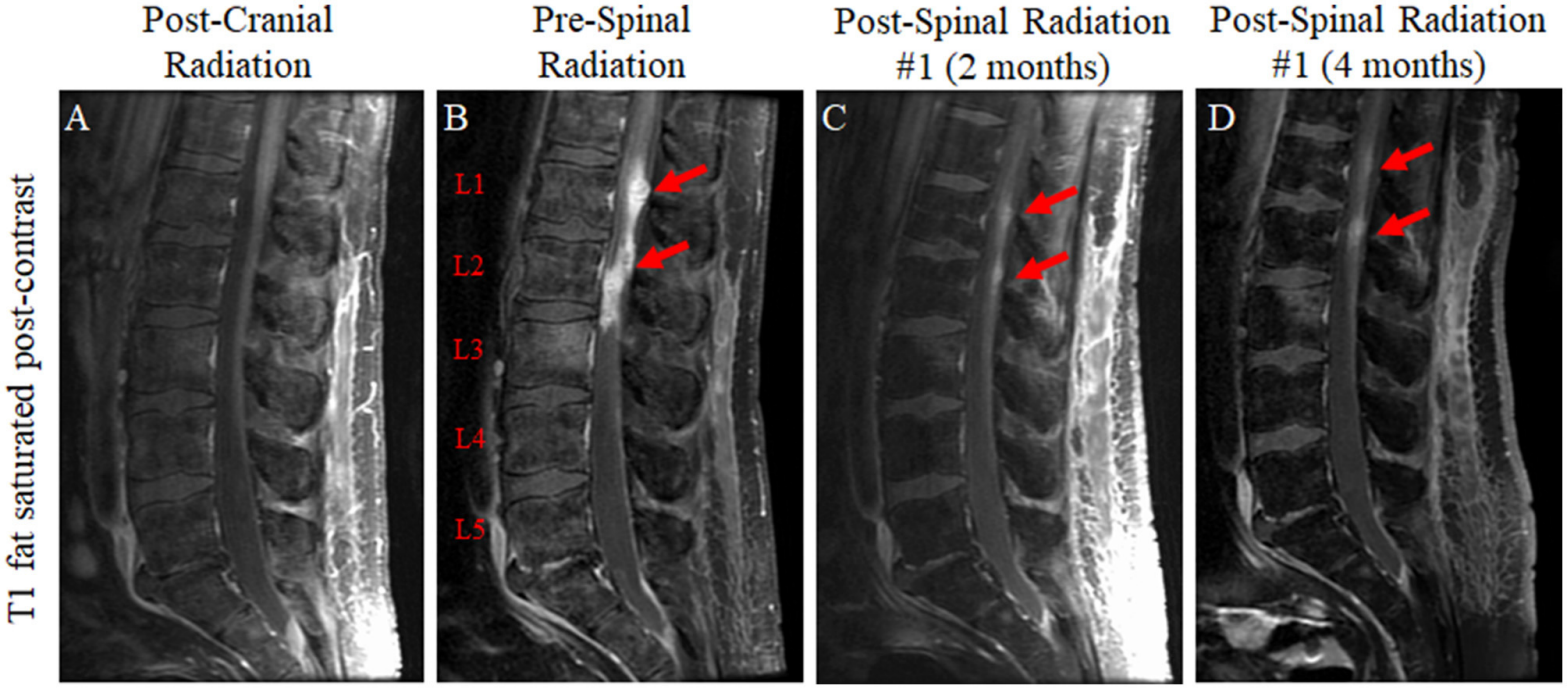
T1-weighted fat-saturated post-contrast sequences of the lumbar spine for the patient in case 2 immediately following radiation to the brain lesion **(A)**; 7 months later following the development of new lower back pain which revealed leptomeningeal drop metastases at the level of T12 extending to the terminus of the thecal sac (T12 to L3 predominant lesions represented graphically) **(B)**; approximately 2 months after the completion of 30 Gy of radiation in 10 fractions to T10-S4 with a significant reduction in lesion size **(C)**; and approximately 4 months after the completion of spinal radiation **(D)**.

Unfortunately, despite the previously unremarkable spine imaging (Figure 4A), the patient developed extensive leptomeningeal drop metastases at the level of T12 extending to the terminus of the thecal sac 7 months after the completion of brain radiation (Figure 4B). T10-S4 was treated with radiation to 30 Gy in 10 fractions with significant disease response observed on MRI 2 months later (Figure 4C). New cervical and thoracic leptomeningeal drop metastases outside the prior radiation field were observed 2 months later in previously non-irradiated regions as well as slightly increased conspicuity of several previously treated lumber lesions, most notably at the L2 level (Figure 4D). As such, C6-T6 was irradiated similarly, and L1-L3 was re-irradiated to an additional 25 Gy in 10 fractions. Other than post-radiation fatigue, the patient continued to maintain his functional status.

Prior to the radiographic evaluation of the re-irradiated spine, while no disease recurrence was seen at the primary site, an MRI of the head 12 months post-initial radiation, unfortunately, demonstrated ependymal disease progression to five sites including in the contralateral brain outside the prior radiation field. Per patient preference, palliative radiotherapy to 25 Gy in 5 fractions was delivered to each lesion with margin coverage. Two months later, an MRI of the head revealed a near-complete response of all five sites of the disease. Unfortunately, an MRI of the spine showed new progression in regions of the thoracic, lumbar, and sacral spinal canal that had not been recently irradiated. In order to preserve the quality of life and given limited meaningful systemic options that would allow this, the patient opted for hospice care. He died 17 months after the completion of the initial radiation without evidence of disease recurrence at the primary site.

## Discussion

Outcomes for high-grade gliomas, including GB, remain poor with limited recent therapeutic advances beyond radiotherapy, TMZ, and alternating electric tumor-treating fields (7, 13). In contrast to most *de novo* GBs in which an inciting etiology is unknown, patients with RIGs have the unique unifying factor of clear prior mutagenic agent exposure. Because of this, it has been suggested that these RIGs may share an underlying molecular signature secondary to their pathogenesis (8, 10, 11). Despite the emergence of the next-generation sequencing use in glioma in recent years to better understand disease biology and evaluate patient candidacy for nontraditional therapeutic options, this testing remains an inconsistent practice, and the relative rarity of RIGs has further limited widespread reporting of characteristic alterations. Here, we add to the limited molecular literature on RIGs with two unique clinical cases demonstrating remarkably unusual complete responses to treatment following subtotal resections on the basis of Response Assessment in Neuro-Oncology (RANO) criteria, a phenomenon not previously reported in RIGs and only rarely observed in GB independent of bevacizumab-mediated pseudoresponse (14–18).

To date, the largest studies of high-grade RIGs were published simultaneously in 2021. DeSisto et al. comprehensively evaluated 32 tumors by DNA methylation profiling as well as both DNA and RNA sequencing (19). Interestingly, when compared to a non-RIG control cohort, this study, indeed, suggested that many of these tumors could be subgrouped together; methylation profiling of 25 of 31 evaluable tissues clustered epigenetically with pediatric receptor tyrosine kinase I (pedGBM-RTK1) and *H3K27M* negative midline high-grade gliomas with an odds ratio of 29.2 for falling into this group compared to non-RIG. This was independent of time from radiation and original pediatric diagnosis. Copy number alterations were more frequently seen in RIGs including Chr 1p loss (40%) and/or 1q gain (50%), Chr 13q loss (40%), Chr 14q loss (40%), *PDGFRA* amplification (36%), *CDKN2A* loss (29%), *BCOR* loss (23%), and *CDK4* amplification (19%) with findings in the surface tyrosine kinase receptor gene, *PDGFRA*, and transcriptional co-repressor with influence on apoptosis, *BCOR*, trending toward and demonstrating significance, respectively. Somatic non-copy number alterations were most often seen in *PDGFRA, TP53, MET*, and *NF1*. Deng MY et al. also conducted genetic and expression profiling of 32 RIGs and similarly found that 91% of tumors had methylation profiling consistent with the pedGBM-RTK1 subtype (8). Further, the most commonly observed alterations were again *PDGFRA* amplification (53%), *CDKN2A/B* loss (66%), *CDK4* amplification (16%), Chr 1p loss (59%) and/or 1q gain (50%), Chr 13q loss (72%), and Chr 14q loss (45%) as well as Chr 6q loss (56%) and *MET* amplification (28%). Somatic non-copy number alterations reported included *PDGFRA, EGFR, TP53, MET, NTRK2, RAF1, BCOR*, and *ATRX*. *TERT* promotor and *IDH1/2* mutations were distinctly absent along with *EGFR* amplification and polysomy 7/monosomy 10.

Beyond these studies, most RIG genomic analyses have been reported in collectives of small cohorts. López GY et al. profiled 12 RIGs and noted frequent alterations in *TP53* (58%), *PDGFRA* (50%), *CDK4* (33%), and *CDKN2A/B* (33%) in addition to less consistent means of MAP kinase pathway activation (11). Significantly aneuploid genomes were seen in the 10 high-grade gliomas. Whitehouse et al. conducted a systematic review in 2021 to better characterize the molecular features of previously reported RIGs (10). Thirty-one reports including 102 unique high-grade RIGs with molecular data were summarized. Again, the most common alterations observed were *PDGFRA* and *CDK4* amplification, *CDKN2A* loss, *PDGFRA* and *TP53* mutations, and chromosomal changes including 1p loss, 1q gain, and 13q loss; the authors concluded that these findings suggested that RIGs were molecularly aligned with the pedGBM-RTK1 subgroup.

The two cases presented here demonstrate molecular similarities with those reported in the literature and further support the unique genomic clustering of RIGs. This genomic clustering raises diagnostic considerations given new histologic and molecular classification criteria for gliomas issued in the recent fifth edition (2021) of the WHO Classification of CNS Tumors. Though many RIGs, including both cases here, possess necrosis and/or microvascular proliferation, which in the context of an IDH-wildtype genomic profile is sufficient to establish a diagnosis of GB based on 2021 classification criteria, they actually possess a molecular signature more consistent with diffuse pediatric-type high-grade glioma (PTHGG), *H3*-wildtype, IDH-wildtype, and WHO grade 4; this is a newly minted tumor class in the fifth edition of the WHO classifications comprising a subset of pediatric high-grade gliomas. Such findings are further exemplified through our cases and literature reports by a general paucity of known GB disease-defining alterations including polysomy 7/monosomy 10, *TERT* promotor mutation, or *EGFR* amplification (20). Indeed, if our cases lacked grade 4 histologic features—vascular proliferation and necrosis—as is sometimes observed in incipient grade 4 gliomas, their histologic and molecular features would be insufficient to establish a diagnosis of GB, IDH-wildtype, based on the 2021 criteria. However, the cases would be compatible on both histologic and molecular grounds with PTHGG, with the caveat that such tumors should not be diagnosed outside pediatric and young adult populations. These findings highlight diagnostic ambiguity in RIG classification with the new criteria. Such unifying molecular events suggest that gliomas with molecular profiles of PTHGG in the context of prior radiation exposure may be best given in their own diagnostic category.

Specifically unique to our cases was the marked response to therapy, something not previously reported. Indeed, survival data for RIGs suggest an aggressive disease with poor outcomes (5, 8, 19). While the role of multiple survival and proliferative pathways has clearly been demonstrated to impact the DNA damage response, nothing unique to the genomic profiles of the two cases provides an indication that either tumor would show increased responsiveness to radiation or TMZ (21–23).

In case 1, mutations were observed in *BRCA1* (*breast cancer type 1*; Q1327R) and *XPC* (*Xeroderma pigmentosum complementation group C*; I165V). While pathogenic alterations in *BRCA1* impede the repair of double-stranded DNA breaks as acquired by ionizing radiation, and *XPC* dysfunction has been shown to increase TMZ-induced DNA damage, neither point mutation correlated with meaningful functional alteration of the resultant protein nor loss of heterozygosity was observed (24, 25). Case 2 revealed a complex tetraploid chromosomal pattern, reported in approximately 11% of GB as an early event in IDH-wildtype GB (26). While no specific pathogenic alterations in DNA damage repair genes were observed in this case, the duplication of the genome has been associated with genomic instability in GB, potentially portending increased sensitivity to radiation (26). Interestingly, despite a lack of reports suggesting increased susceptibility to DNA-damaging agents, a subset of RIGs harbors decreased transcriptional expression of DNA repair gene products, thus implicating a potential mechanism of improved therapeutic sensitivity in some RIGs (19). Identification of such lesions would require RNA sequencing and/or proteomics which are not yet routine in practice and have technical limitations.

Importantly, response to therapy was independent of *MGMT* promotor methylation status as there was discordance of this biomarker between the two cases. In fact, given the patient's initial poor performance status and the lack of *MGMT* promotor methylation in case 2, TMZ was not offered, indicating that the significant response to therapy was not dependent on this known favorable marker nor the administration of chemotherapy. The perioperative appearance of both tumors and the gross pathologic characterization of tissue were reported to be consistent with the typical appearance of a GB without unique or unexpected features.

Our two cases demonstrate the complexity of managing RIGs and predicting outcomes. Despite remarkable complete responses at the sites of the original disease following early postoperative regrowth, one patient developed early parenchymal necrosis in the setting of prior radiation exposure; the other developed overwhelming leptomeningeal spread that, while also markedly responsive to radiation, ultimately exhausted safe treatment options. Sequencing and microarray evaluation of these cases demonstrate similarities with a clustered analysis of other RIGs and add to the molecular literature while contrasting outcomes. This clearly demonstrates that the future evaluation of the tissue will require additional diagnostic testing to parse out disease biology for the prediction of treatment responses. Given the rarity of RIGs, as well as the currently relatively limited case series in the literature compiling corresponding molecular, treatment, and outcome data, a collaborative multi-institutional clinical and pathological review of RIGs would represent an important opportunity to gain meaningful insight into this understudied disease moving forward.

## Data availability statement

The datasets presented in this article are not readily available because of ethical and privacy restrictions. Requests to access the datasets should be directed to the corresponding author.

## Ethics statement

Written informed consent was obtained from the individual(s) for the publication of any potentially identifiable images or data included in this article.

## Author contributions

PG, JH, and HR: acquisition of material. PG, JH, SH, and HR: direct patient management/data implementation. PG, JH, DD, SH, RJ, and HR: analysis of data/information and interpretation of data/information. All authors contributed to the article and approved the submitted version.
